# Unusual Case Report of Headache in 10-Year-Old Female Child

**DOI:** 10.7759/cureus.53590

**Published:** 2024-02-04

**Authors:** Sudesh Kumar, samragnee mondal, Roshan Kumar

**Affiliations:** 1 Pediatrics, Mata Gujri Memorial Medical College, Kishanganj, IND; 2 Medicine, Mata Gujri Memorial Medical College, Kishanganj, IND

**Keywords:** migraine, venography, sigmoid, fundoscopy, hypoplasia

## Abstract

According to the literature, transverse sinus hypoplasia is not a normal variant and has a serious potential effect on cerebral blood flow. We are presenting a rare case of chronic headache due to severe hypoplasia of the left transverse and sigmoidal sinus. A 12-year-old female girl was admitted with a complaint of gradual progressive severe headache, throbbing in nature, confined to a bitemporal and frontal region in the last 4-5 months. Headache is not associated with fever, vomiting, photophobia, or vision problems. The child had no history of recurrent running nose, refractory vision, ear discharge, head trauma, exanthemata rash, or any drug history. On examination, the child was conscious and oriented. Vital signs are normal. The child was neurologically normal and had no focal signs. Other systemic examinations were normal. Based on History and examination, differential diagnosis was made, like Pseudo tumor cerebri, migraine, deep vein sinus thrombosis, and functional and Posterior fossa tumor. The child had normal routine investigations like complete blood count, electrolyte, and D-dimer. The fundoscopy was normal. In MRI, brain hypoplasia of the left transverse and sinusoidal sinus was suspected and confirmed by MRI venography. Thus, for any patient in an emergency with a chronic headache without focal signs and normal fundoscopy, one deferential should be considered for transverse and sigmoid sinus hypoplasia.

## Introduction

Drainage of blood from the cranium and brain through a network of venous channels known as dural venous sinuses, which have no valve and musculature, unlike a systemic vein. The anatomical asymmetry of the transverse sinus is common, and 20-39% of cases have been associated with unilateral hypoplasia or aplasia [1,2]. According to the literature, transverse sinus hypoplasia is not a normal variant and has a serious potential effect on cerebral blood flow [3-6]. Transverse and sigmoidal sinus aplasia or hypoplasia is associated with intracranial hypertension without papilledema, which is one of the risk factors for chronic headaches [7]. We are presenting a rare case of chronic headache due to severe hypoplasia of the left transverse and sigmoidal sinus, which is rarely documented in pediatric age.

## Case presentation

A 12-year-old female girl was admitted with a complaint of gradual progressive intermittent severe headache, throbbing in nature, confined to the bitemporal and frontal region for 4-5 months. Headache is not associated with fever, vomiting, photophobia, or vision problems. The child had no history of recurrent running nose, refractory vision, ear discharge, head trauma, exanthemata rash, or any drug history. The headache was not relieved by the paracetamol drug. On examination, the child is conscious and oriented. Vital signs are normal. The child was neurologically normal and had no focal signs. Other systemic examinations were normal. Based on History and examination, differential diagnosis was made, like Pseudo tumor cerebri, Migraine, Deep vein sinus thrombosis, and Functional and Posterior fossa tumor.

**Figure 1:**
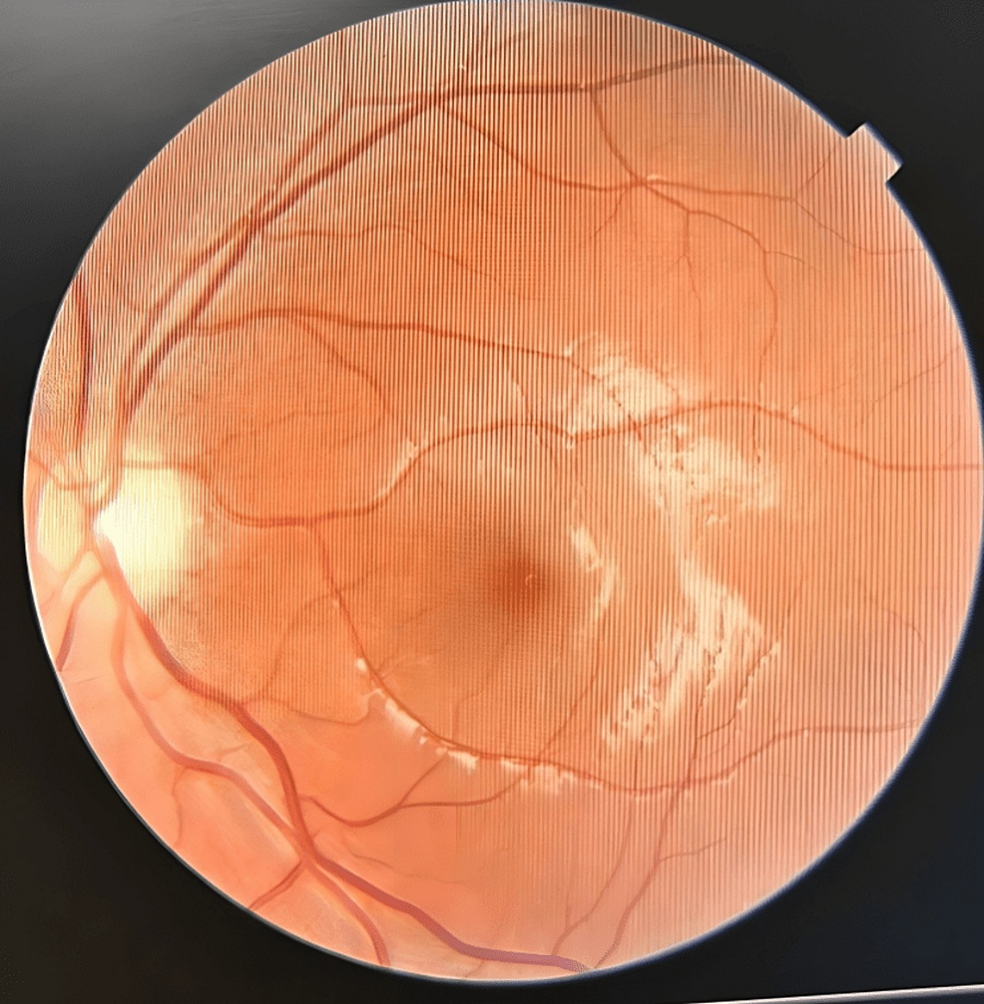
Fundoscopy shows no papilloedema

**Figure 2:**
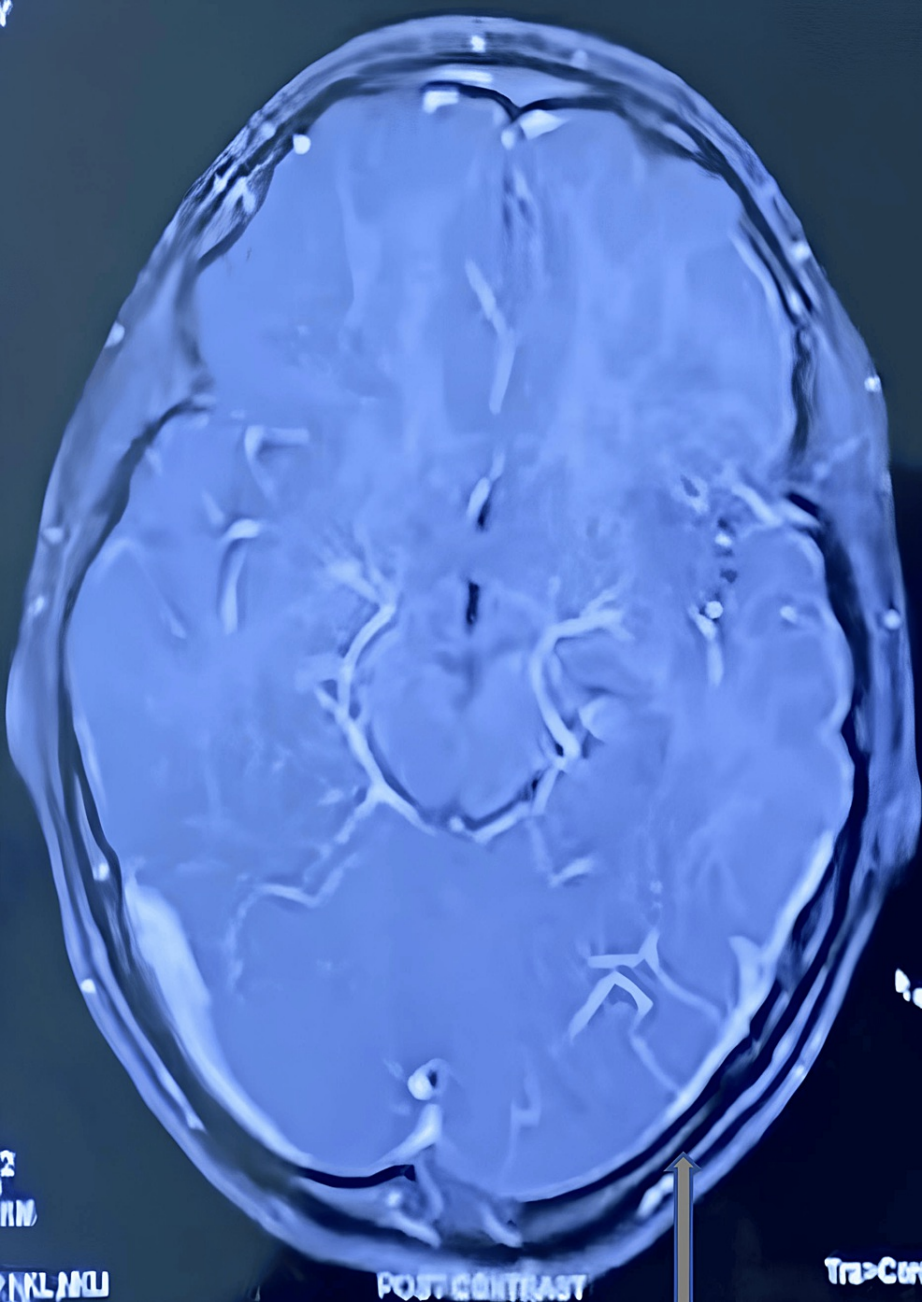
Shows hypoplasia of left transverse and sigmoid sinus hypoplasia

**Figure 3:**
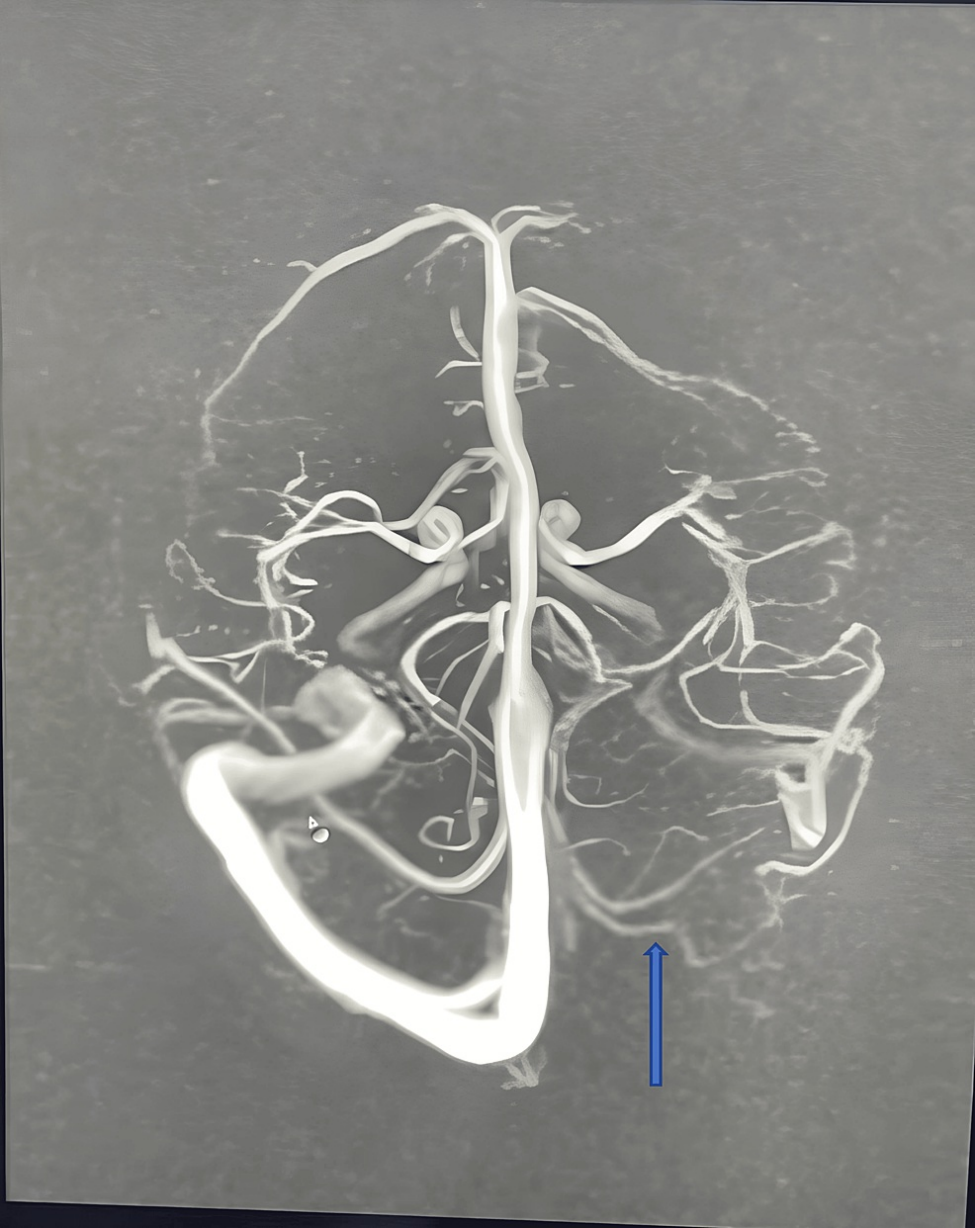
Showing left transverse and sigmoid sinus not visualized

In an investigation in complete blood count (CBC)- hemoglobin (Hb) 11.5 gr/dl, total leucocyte count (TLC) 9700, neutrophil (N) 60%, lymphocyte (L): 31%, C reactive protein (CRP): <6 mg/L, sodium (Na): 135 mEq/L, potassium (K): 4.49mEq/L, Calcium (Ca): +9.14 mg/dl. Kidney function test (KFT), liver function test (LFT), Prothrombin time (PT), and activated partial thromboplastin time were normal. HIV and Hepatitis surface antigen serology was normal. The D-dimer level was within normal limits. X-ray chest and EEG report were normal. No papilledema or optic atrophy (Fig 1) was noted in fundoscopy. An MRI brain showed that the left transverse and sigmoid sinus was not visualized - likely atretic/severe hypoplasia and the right transverse and sigmoid jugular system is dominant (Fig 2), which was confirmed by MRI angiography (Fig 3) and the final diagnosis was confirmed as severe left transverse and sigmoid sinus hypoplasia. The patient required a surgical process as a treatment, so the patient was transferred to a neurosurgeon.

## Discussion

Severe left transverse and sigmoid sinus hypoplasia was diagnosed in my case by MR venography, which presented with severe headache, not responding with symptomatic treatment. Fofi L et al. [1] studied the transverse sinus morphology in 83 patients by MR venography and found nervous disease; hypertension, migraine, and anxiety have been reported more commonly in patients with transverse sinus aplasia and hypoplasia. Lin CJ et al. [2] showed that the ipsilateral hypoplastic transverse sinus was associated with prolonged circulation and that inadequate venous drainage might play a role in impaired autoregulation. Wilson MH et al. [4] showed that restriction in cerebral venous outflow may lead to headaches when hypoxia-related increased arterial flow occurs. De Simone R et al. [6] showed that a neurological series of intracranial hypertension without papilledema had been found in about one-half of a chronic primary headache with poor response to symptomatic treatment and in all abnormal MR Venography. Alper F et al. studied the importance of anatomical asymmetries of the transverse sinus in 105 patients by MR venography that found left sinus aplasia in 20% and left sinus hypoplasia in 39%[7]. Bono F et al. [8] showed short-term CSF pressure monitoring through a lumber needle and found abnormal pressure waves and elevated CSF pressure in most headache sufferers with bilateral transverse sinus stenosis. Fofi L et al. [9] showed that advanced MR venography disclosed transverse sinus asymmetry in as many as 50.6% of patients with chronic migraine. In this case report, we presented a patient with severe left-sided transverse and sigmoid sinus hypoplasia, which is rarely documented in pediatrics.

## Conclusions

In this case report, we presented a patient with severe left-sided transverse and sigmoid sinus hypoplasia, which is rarely documented in pediatrics. Thus, for any patient who comes in an emergency with a chronic headache without focal signs and normal fundoscopy, one deferential should be considered for transverse and sigmoid sinus hypoplasia because Transverse sinus anatomical variation is not uncommon and should be considered before venous sinus thrombosis.
